# Preoperative education in hip and knee arthroplasty patients in Bloemfontein

**DOI:** 10.4102/sajp.v74i1.436

**Published:** 2018-05-29

**Authors:** Roline Y. Barnes, Karen Bodenstein, Nadia Human, Jacques Raubenheimer, Jodri Dawkins, Carmen Seesink, Jonè Jacobs, Jolien van der Linde, Ruan Venter

**Affiliations:** 1 Department of Physiotherapy, University of the Free State, South Africa; 2 Department of Biostatistics, University of the Free State, South Africa

## Abstract

**Background:**

Total hip arthroplasty (THA) and total knee arthroplasty (TKA) are frequently performed surgeries worldwide. Preoperative education enhances patient physiotherapy management and satisfaction and should be tailored to patients’ educational needs. Limited research is available regarding the preoperative educational needs for these patients.

**Objectives:**

To determine the extent of preoperative education received and the preoperative educational needs of patients undergoing THA and TKA.

**Method:**

A structured interview utilising a self-developed questionnaire was used and included questions exploring preoperative education, educational needs, method of education and health care professional providing education. A total of 14 THA and 36 TKA patients, 2–4 days post-operatively at private hospitals in Bloemfontein, were conveniently sampled.

**Results:**

All participants had arthroplasties because of osteoarthritis. All participants with THA and 35 (98%) participants with TKA received preoperative education from orthopaedic surgeons, and 8 (57%) participants with THA and 9 (25%) participants with TKA received preoperative education from physiotherapists. Education was mostly given as pamphlets months before the surgery. Participants received the least amount of information regarding exercises, especially preoperative exercise, pain relief and activities of daily living.

**Conclusion:**

This study highlights the need for improvement in patient engagement and education, together with enhanced health care practitioner communication and collaboration. Patient centeredness and individualised THA and TKA preoperative education programmes are recognised as a necessary attribute of quality health care and can lead to improved THA and TKA outcomes. The importance of exercise as part of preoperative interprofessional education in the management of THA and TKA should be emphasised as exercise is the cornerstone for rehabilitation of THA and TKA.

**Clinical implications:**

This study aimed to emphasise the importance of tailored preoperative education for THA and TKA patients to improve patient outcomes.

## Introduction

Total hip arthroplasty (THA) and total knee arthroplasty (TKA) are amongst the most frequently performed surgeries in developed (Cram et al. 2012; Learmonth, Young & Rorabeck 2007) and developing countries (Jain et al. 2004). Arthroplasty is an effective intervention to reduce pain and improve quality of life and functional limitations in individuals living with symptomatic degenerative hip and knee disorders (Carr et al. 2012; Pivec et al. 2012; Soever et al. 2010). In South Africa, THA and TKA patient admissions in the private sector have increased by 31% and 53%, respectively, and the current private health care costs have escalated by approximately 300% (Ngoepe 2016) in the past decade. Because of the escalation in current health care costs, it seems reasonable to hypothesise that there should be a shift in patient management in order to improve patient outcomes and reduce health care costs. Preoperative education might be the essential non-surgical element to facilitate the shift in patient management and empower patients to make the best possible health care decisions (Fink et al. 2013).

Preoperative education refers to an educational intervention delivered to patients prior to surgery, consisting of surgical information, pain management and preoperative exercises (Bernier et al. 2003; Louw et al. 2012). According to Sjöling et al. (2003) and Kagan and Bar-Tal (2007), preoperative anxiety impacts THA and TKA patients’ perception of pain and coping and has an influence on patients’ functional outcomes (Kagan & Bar-Tal 2007; Sjöling et al. 2003).

Associated post-operative complications including infection and deep venous thrombosis are less frequent in patients receiving THA and TKA preoperative education compared with patients receiving no education (Wylde et al. 2011). Furthermore, according to Yoon et al. (2010), preoperative education reduces the length of hospital stay for patients undergoing THA or TKA (Yoon et al. 2010), which has an added financial benefit for the individual (Clarke et al. 2012). According to Andrawis et al. (2015), preoperative education can equip patients with a realistic expectation of the surgical outcomes, improve patient satisfaction regarding surgical outcomes and enhance patient motivation (Andrawis et al. 2015).

A major concern identified by Kruzik as early as 2009 is that preoperative education provided by health care practitioners (HCPs) for THA and TKA is not directed at the specific individual and that the individual’s educational needs are often unmet (Kruzik 2009 McDonald et al. 2014). It is advisable that preoperative education should be tailored to suit the specific individual (McDonald et al. 2014).

Limited research is available regarding the preoperative educational needs for THA and TKA patients, and no research on this topic could be found in a South African context. The aim of this study was thus to determine the extent of preoperative education currently provided to elective THA and TKA patients in the private sector in Bloemfontein, South Africa, and to determine the preoperative educational needs expressed by these patients.

## Methodology

A quantitative descriptive study was undertaken. Data were collected by means of a structured interview utilising a self-developed questionnaire. The study population consisted of post-operative patients who underwent elective THA or TKA between February and May 2016. Convenience sampling was used, because of the limited anticipated number of participants after a pilot study. A pilot study was conducted according to the procedure of the main study in the surgical orthopaedic ward of three private hospitals. Five participants, 2–4 post-operative days following THA and TKA, were interviewed. The duration of the interview stipulated in the information letter was amended after the pilot study. During the pilot study, the authors noted that participants who had previous THA or TKA had received previous preoperative education and had some knowledge regarding the surgical experience. A history of previous THA or TKA was thus added to the exclusion criteria. One of the sites identified for the study was also excluded as the number of possible participants was limited. The data from the pilot study were included in analysis as no major changes were made to the study procedure, except one participant who was excluded because of a previous arthroplasty. Participants signed informed consent prior to being involved in the study.

The self-developed questionnaire was based on the Patient Learning Needs Scale (PLNS) (Bubela et al. 1990) and the Canadian Clinical Checklist (Soever et al. 2010). The correlation of the subscale to total scores in the PLNS ranged from 0.69 to 0.85, whereas the inter-correlation of the subscale scores was 0.42 to 0.71. The internal consistency is high with an alpha coefficient of 0.95. Sets of items in each of the seven factors have alpha coefficients ranging from 0.69 to 0.88 (Bubela et al. 1990). The Canadian Clinical Checklist was compiled from a qualitative study, and therefore, no validity or reliability values were provided in the article. The questionnaire explored participants’ demographic information, reason for surgery and preoperative education provided to the participants in the following categories: surgery and medication, general hospital care, precautions, activities of daily living (ADL), home care and family advice and, lastly, exercises. The questionnaire further explored participants’ needs for preoperative education according to the previously mentioned five categories, the preoperative education delivery method, as well as the source and time frame in which the education was received. The self-developed questionnaire did not go through any validity and reliability processes as part of this study.

The study was conducted in private hospitals in Bloemfontein. Elective THA or TKA patients, 2–4 days post-operatively, older than 18 years of age and able to understand and converse in English or Afrikaans were included in the study. Patients who had undergone the arthroplasty secondary to trauma or who had undergone a previous THA or TKA were excluded. Authors J.D., J.J., R.V., C.S. and J.v.d.L. conducted the interview in either English or Afrikaans according to the participant’s language preference. They noted each participant’s answers, and after the completion of the interview, an opportunity was provided to participants to review their completed questionnaires to ensure that the answers were a true reflection of what they had said. Both J.D. and R.V. coded the completed questionnaires and the data in three separate Excel spreadsheets after which the data were compared by C.S., J.J. and J.v.d.L. Compared and revised spreadsheets were analysed by the Department of Biostatistics, University of the Free State. Descriptive statistics, namely frequencies and percentages, were calculated for categorical data. All electronic data were kept safe by a password-protected document on a storage CD.

### Ethical considerations

Ethical clearance for this study was granted by the Ethics Committee of the Health Sciences Faculty of the University of the Free State (75/2015).

## Results

A total of 14 THA and 36 TKA participants were included in this study, with a mean age of 64 years. Just more than half of the participants (26, 52%) were male, and 42 (84%) participants were Afrikaans speaking. The main reason indicated for participants’ arthroplasties was osteoarthritis (THA: 13, 93%; TKA: 31, 86%), with the most common preoperative symptom being pain (THA: 13, 93%; TKA: 34, 94%).

All the THA participants received education verbally and 11 (79%) participants as a pamphlet. The majority of TKA participants 32 (89%), received a pamphlet, and 14 (39%) TKA participants received education verbally. Six (43%) THA participants received the education months prior to surgery, while 21 (57%) TKA participants received the education months prior to surgery and 14 (39%) TKA participants received the education, weeks prior to surgery. All of the THA and 35 (98%) of the TKA participants indicated that they received their preoperative education from an orthopaedic surgeon with only 8 (57%) of the THA and 9 (25%) of the TKA participants receiving preoperative education from a physiotherapist.

In the surgery and medication section of the questionnaire, all of the THA participants (Table 1) and 33 (92%) of the TKA participants (Table 2) received preoperative education on possible post-operative complications, namely deep venous thrombosis, and 43 (86%) of both THA and TKA participants received preoperative education regarding infection as a complication post-operatively. Thirteen (93%) THA participants also received preoperative education on loosening of the prosthesis (Table 1). Four (30%) THA and seven (19%) TKA participants perceived the information on post-operative information as being insufficient.

**TABLE 1:** Education received on potential post-operative complications after total hip arthroplasty (*n* = 14).

Topic	No education received *n* (%)	Education received preoperatively *n* (%)	Education received only post-operatively *n* (%)	Education received pre- and post-operatively *n* (%)
Blood clots	0 (0)	14 (100)	0 (0)	0 (0)
Pulmonary embolism	5 (36)	9 (64)	0 (0)	0 (0)
Infection	2 (14)	12 (86)	0 (0)	0 (0)
Infection leading to removal of implant or implants	5 (36)	9 (64)	0 (0)	0 (0)
Loosening of the prosthesis	1 (7)	13 (93)	0 (0)	0 (0)
Difference in leg lengths	7 (50)	7 (50)	0 (0)	0 (0)
Dislocation	2 (14)	10 (72)	1 (7)	1 (7)
Joint stiffness	7 (50)	7 (50)	0 (0)	0 (0)

**TABLE 2:** Education received on potential post-operative complications after total knee arthroplasty (*n* = 36).

Topic	No education received *n* (%)	Education received preoperatively *n* (%)	Education received only post-operatively *n* (%)	Education received pre- and post-operatively *n* (%)
Blood clots	1 (3)	33 (92)	2 (5)	0 (0)
Pulmonary embolism	9 (25)	26 (72)	1 (3)	0 (0)
Infection	3 (8)	31 (86)	2 (6)	0 (0)
Infection leading to removal of implant or implants	7 (19)	28 (78)	1 (3)	0 (0)
Loosening of the prosthesis	15 (42)	20 (56)	1 (3)	0 (0)
Dislocation	14 (39)	22 (61)	0 (0)	0 (0)
Joint stiffness	7 (19)	29 (81)	0 (0)	0 (0)

Nine (64%) THA participants and 35 (97%) TKA participants received preoperative education on wearing pressure stockings to prevent deep venous thrombosis post-operatively. The use of a triangular cushion to prevent internal rotation and dislocation of the hip when lying in bed or sleeping was discussed preoperatively with 7 (50%) THA participants.

Precautions that participants had to adhere to post-operatively to prevent dislocation of the prosthesis were discussed preoperatively with 6 (43%) THA participants and 12 (33%) TKA participants (Table 3).

**TABLE 3:** Education received on post-operative precautionary measures after total hip arthroplasty and total knee arthroplasty.

Precautionary measures	No education received *n* (%)	†Education received preoperatively *n* (%)	Education received only post-operatively *n* (%)
**THA (*n* = 14)**			
Not allowed more than 90° hip flexion	0 (0)	6 (43)	8 (57)
No adduction across midline	0 (0)	6 (43)	8 (57)
No hip internal rotation	0 (0)	6 (43)	8 (57)
Combination of these movements	0 (0)	6 (43)	8 (57)
**TKA (*n* = 36)**			
Not allowed to hunch	10 (28)	12 (33)	14 (39)
Not allowed to kneel	10 (28)	12 (33)	14 (39)
Not allowed to pivot	11 (31)	9 (30)	14 (39)

THA, total hip arthroplasty; TKA, total knee arthroplasty.

† , includes participants who received education pre- and post-operatively.

Education on ADL such as the correct way to get in and out of bed, climb stairs and lift an object was mostly provided post-operatively (Tables 4 and 5). Among the THA participants, 3 (21%) never received education on lifting an object or getting in and out of a car (Table 4), whereas 12 (33%) and 15 (42%) of the TKA participants never received education on these topics (Table 5). Information on how to use a walking aid was provided to 8 (57%) THA participants preoperatively and to 19 (53%) TKA participants post-operatively. One (7%) THA participant and five (14%) TKA participants never received information on how to use a walking aid (Tables 4 and 5). Education on how long to use the walking aid, when participants could return to driving and participating in sport was mostly received preoperatively in both groups of participants (Tables 4 and 5). Two (15%) THA and eight (22%) TKA participants perceived the information on ADL to be insufficient.

**TABLE 4:** Education received on return to and the correct way to perform ADL after total hip arthroplasty (*n* = 14).

Activity	No education received *n* (%)	†Education received preoperatively *n* (%)	Education received only post-operatively *n* (%)
Sit	1 (7)	4 (29)	9 (64)
Stand	1 (7)	4 (29)	9 (64)
Lie down or sleep	0 (0)	5 (36)	9 (64)
Walk	0 (0)	5 (36)	9 (64)
Lift objects	3 (21)	4 (29)	7 (50)
Get in and out of the bed	0	4 (29)	10 (71)
Climb stairs	1 (7)	4 (29)	9 (64)
Get in and out of the car	3 (21)	4 (29)	7 (50)
How to use a walking aid	1 (7)	8 (57)	5 (36)
How long to use the walking aid	1 (7)	9 (64)	4 (29)
Return to driving	2 (14)	10 (72)	2 (14)
Return to sport	5 (36)	8 (57)	1 (7)

† , includes participants who received education pre- and post-operatively.

**TABLE 5:** Education received on return to and the correct way to perform activities of daily living after total knee arthroplasty (*n* = 36).

Activity	No education received *n* (%)	†Education received preoperatively *n* (%)	Education received only post-operatively *n* (%)
Sit	6 (17)	7 (19)	23 (64)
Stand	6 (17)	7 (19)	23 (64)
Lie down or sleep	2 (6)	8 (22)	26 (72)
Walk	2 (6)	8 (22)	26 (72)
Lift objects	12 (33)	6 (17)	18 (50)
Get in and out of the bed	4 (11)	6 (17)	26 (72)
Climb stairs	5 (14)	6 (17)	25 (69)
Get in and out of the car	15 (42)	5 (14)	16 (44)
How to use a walking aid	5 (14)	12 (33)	19 (53)
How long to use the walking aid	9 (25)	18 (50)	9 (25)
Return to driving	5 (14)	26 (72)	5 (14)
Return to sport	7 (19)	24 (67)	5 (14)

† , includes participants who received education pre- and post-operatively.

In the section on home care and family advice, participants were asked whether they were informed about adaptations to be made in their home environment after surgery as well as the need for assistance from someone for the first 2 weeks after surgery. In the THA group, preoperative education on the use of a high chair with arm rests was provided to 12 (86%), a raised toilet seat to 11 (79%), the use of assistive devices for ADL to 7 (50%) and someone to assist them to 9 (64%). However, 9 (64%) of the THA participants did not receive any education on removing tripping hazards and clutter to prevent a fall. In the TKA group, 28 (78%) participants did not receive any education on removing tripping hazards and clutter and 19 (53%) participants were not informed that they would need the assistance of someone for the first 2 weeks after surgery.

Participants had to indicate whether they received education on exercises to be performed preoperatively and post-operatively. Eleven (76%) THA participants and 27 (74%) TKA participants did not receive any education regarding preoperative exercises. Nine (63%) THA participants and 28 (78%) TKA participants received information on post-operative exercises post-operatively. In the THA group, eight (57%) participants did not receive any information on pain relief, six (43%) participants received information preoperatively and no participants received information on pain relief post-operatively. In the TKA group, 17 (47%) participants did not receive any information on pain relief, 14 (39%) participants received information preoperatively and only 5 (14%) participants received information on pain relief post-operatively.

## Discussion

The majority of the participants in our study received preoperative education in the form of a written pamphlet. Written pamphlets are an effective preoperative education delivery method because they enhance patient information retention as patients can refer to the information at a later stage if needed. It is of importance that written preoperative education is provided at a level suitable for the patient (Gaston & Mitchell 2005) and is tailored according to the individual’s educational needs (Becker et al. 2007; Raynor et al. 2007). McMurrey et al. (2006) found that written pamphlets combined with verbal explanation represent the most effective delivery method for providing health education. Therefore, it is recommended that a combined method should be used to provide preoperative education to patients (McMurrey et al. 2006).

Most of the participants received preoperative education weeks and even months prior to surgery. Although it may be beneficial to provide patients with preoperative education prior to hospitalisation with the aim of decreasing anxiety levels (Hughes 2002), a study in 2007 found that providing the education during hospitalisation is more effective, as patients may be more open to education in the hospital setting (Merriam & Kim 2009). It seems that there is no optimal time frame to provide preoperative education (Hughes 2002; Merriam & Kim 2009). To be noted is that both of the above studies emphasised the importance of providing preoperative education at a time preferred by the patient.

Studies conducted in Canada have shown that physiotherapists working in orthopaedic wards provided more preoperative education and exercise prescription to patients compared to orthopaedic surgeons (Aiken et al. 2007; MacKay et al. 2009), as movement and exercise form an integral part of physiotherapy management (MacKay et al. 2009). The majority of the participants in our study received preoperative education from orthopaedic surgeons, whereas only a small number of participants received preoperative education from physiotherapists. This is a concern as studies clearly indicate the importance of physiotherapy education for preoperative THA and TKA patient management (Czyżewska et al. 2014; Huffsmith & Liu 2014; Jäppinen et al. 2015). No provision was made in the questionnaire in this study to differentiate between the type of information provided by the orthopaedic surgeons and physiotherapists.

The small number of participants receiving preoperative education from a physiotherapist may be the result of patients not being referred by orthopaedic surgeons routinely to physiotherapists before surgery, according to Czyżewska et al. (2014), and the fact that some orthopaedic surgeons may lack confidence in preoperative physiotherapy (Westby & Beckman 2010). According to McCallum (2010) and Odebiyi et al. (2010), a further reason for participants not receiving preoperative education from a physiotherapist is that other members of the HCP team and patients have inadequate knowledge regarding the scope of practice of physiotherapists. Ryan et al. (2005) state that most other members of the HCP do not receive formal training regarding the scope of physiotherapy practice or the benefits of physiotherapy management. These findings suggest that HCPs could lack understanding regarding the importance of the physiotherapist’s role in the preoperative management of THA and TKA patients. Physiotherapists are responsible in all spheres of practice to educate HCPs and advocate the role and scope of physiotherapists as members of the inter-professional team, starting at entry level professional education.

It could thus be hypothesised that a lack of knowledge of orthopaedic surgeons regarding the scope of physiotherapy practice and the benefits thereof could have influenced the surgeon’s lack of preoperative physiotherapy referral. Suter et al. (2009) emphasised that understanding and appreciating the role of each HCP in patient management can improve patient outcomes (Suter et al. 2009). Marten et al. (2014) found that the cost of pre- and post-surgical rehabilitation services and the large number of medical aid schemes in South Africa, which tend to limit the number of rehabilitation sessions, influence the number of referrals for preoperative physiotherapy treatment. It is also stated by Marten et al. (2014) that ineffective provider payment mechanisms may negatively impact the provision of health care services such as preoperative physiotherapy (Marten et al. 2014).

The preoperative educational needs of the participants in a study conducted in Turkey were most evident in the ADL and surgery and medication sections, which include potential post-operative complications (Polat et al. 2014). In our study, it is evident that most of the participants perceived education as sufficient as a large number of THA and TKA participants received preoperative education regarding potential post-operative complications. However, eight TKA and four THA participants received no education regarding various complications. To be noted is that no specific question was posed to participants regarding their understanding of the importance of exercises and activity levels in preventing complications, but it is concerning that a quarter of participants did not receive any education on post-operative complications.

Participants in a study by Soever et al. (2010) expressed a need for additional education in the home care and family advice section. In our study, the TKA group expressed a need for more information on tripping hazards and clutter as well as the need for assistance during the first two weeks after discharge. It seems that participants may have felt that more education in the home care section could enable them to cope better at home after discharge from hospital. This is supported by Orpen and Harris (2010), who found that patients scheduled for THA or TKA experienced less preoperative and post-operative anxiety when receiving preoperative education on home care, as they felt more confident when returning home.

All the THA participants received education on precautionary measures, but mostly received the information post-operatively. Providing the information on precautionary measures preoperatively would assist physiotherapists with the management of patients post-operatively, by emphasising adherence to post-operative precautionary measures preoperatively. The responsibility for providing THA and TKA patients with information regarding precautions in the United States falls solely on physiotherapists (Prouty et al. 2006). In our study, no education regarding precautionary measures was received by 10 TKA participants. This is a concern, as the risk for potential dislocation of the arthroplasty increases if participants are not made aware of the precautions or how to take care of their arthroplasty (Sharma, Morgan & Cheng 2009).

The exercise section was the section in which participants received the least preoperative education. Studies have clearly shown the efficacy and advantages of exercises prior to THA or TKA as well as the numerous positive effects preoperative exercises have on the outcomes of the patient (Huang, Chen & Chou 2011; Matassi et al. 2014; Swank et al. 2011; Villadsen et al. 2014). Preoperative exercises improve the psychological health, social life and quality of life of the patient (Czyżewska et al. 2014). During interviews in studies conducted by Villadsen et al. (2014) and Topp et al. (2009), participants expressed a lack of interest in preoperative exercises, as their main concern was pain relief, clearly indicating that participants were unaware that preoperative exercises might actually reduce their preoperative and post-operative pain. Our study showed that both the THA and TKA participants’ most common preoperative symptom was pain, whereas the majority of the participants indicated that they did not receive any information regarding pain relief pre- or post-operatively. As pain relief forms a part of physiotherapy management, this could be because of the fact that only a small percentage of participants received preoperative education from a physiotherapist.

Czyżewska et al. (2014) explored the effects of preoperative physiotherapy in patients awaiting THA, and 83% of the control group expressed an interest in preoperative physiotherapy if given the opportunity. This is an important finding, as these participants realised the beneficial outcomes of preoperative physiotherapy. This also highlights the importance of physiotherapists educating HCPs and patients regarding the benefits and importance of preoperative exercises, thereby giving patients the opportunity to become role players in their own health outcomes. Communication across members of interdisciplinary care teams has been recognised as a critical element for successful patient management and outcomes. Inter-professional collaboration (IPC) is a model of health care that utilises numerous professional skills to provide the best patient-centred care (Pelling et al. 2011) and should be utilised when compiling preoperative education programmes for patients, as no single disciplinary skill set can fulfil all of the educational needs of patients (Pape et al. 2013). Inter-professional collaboration should be emphasised during undergraduate training, as well as in continuing professional development courses, in order to establish effective health care for all patients.

## Limitations of this study

It is possible that participants’ level of health literacy which in essence affects their ability to understand and retain health care education could have influenced the participants’ understanding of the questions posed during the interview, but this was not determined here. Participants were not specifically asked during the interview whether they had read the pamphlets. This might have influenced the results, as they may have received the education without reading it and therefore perceived it as being insufficient. Only participants who were literate in either English or Afrikaans were included. Our interviews were conducted post-operatively, so possible confusion between pre- and post-operative education could have been experienced by participants, but they were reminded that the questions only referred to preoperative education.

## Recommendations

A follow-up study could be conducted to determine the perception of participants regarding the importance of preoperative exercises prior to arthroplasty, clarifying the lack of interest regarding preoperative exercises.

## Conclusion

As this is one of the first studies conducted in South Africa regarding preoperative education received and the educational needs of arthroplasty patients, several findings are worthy of emphasis. The results highlighted the need for improvement in patient engagement and education, together with enhanced HCP communication and collaboration. Patient centeredness is increasingly being recognised as a necessary attribute of quality health care and can lead to improved THA and TKA outcomes. Therefore, individualised THA and TKA patient preoperative educational needs should be considered and addressed when compiling preoperative educational material.
